# Teachers' Perceptions of Gambling‐Related Risks Among Students With Special Educational Needs: An Exploratory Study

**DOI:** 10.1111/cch.70320

**Published:** 2026-07-14

**Authors:** Megan Dobson, Emily Arden‐Close, Constantina Panourgia

**Affiliations:** ^1^ School of Psychology Bournemouth University Poole UK

**Keywords:** adolescence, gambling‐related risks, qualitative methods, special educational needs (SEN), teachers' awareness

## Abstract

**Background:**

The prevalence of gambling among adolescents is rising, yet teachers often lack awareness of its associated risks. Special educational needs (SEN) students may experience increased vulnerability to gambling‐related harms, although it remains unclear whether teachers recognise this.

**Methods:**

To explore this issue, 15 UK mainstream secondary school teachers were recruited through purposive sampling and participated in online semi‐structured interviews exploring their perceptions of gambling behaviours among typically developing and SEN students. Interviews were audio‐recorded, transcribed verbatim and analysed using an exploratory interpretive approach.

**Results:**

Reflexive thematic analysis identified three themes: *understanding of gambling‐related risks*, *SEN students' vulnerability* and *secondary concern*. Teachers described gambling as a potential risk and perceived SEN students to be more vulnerable, citing cognitive and social deficits as contributing factors. However, gambling was generally viewed as less serious than other risky behaviours, such as substance use. Participants reported that limited training and knowledge contributed to gambling being deprioritised relative to other concerns, leaving them feeling underprepared to address gambling. Although participants perceived SEN students as particularly vulnerable, many expressed low confidence in recognising and responding to gambling‐related risks.

**Conclusions:**

This study underscores the need for targeted professional development to challenge misconceptions and strengthen teachers' competence in addressing gambling‐related risks, particularly in supporting SEN students who may experience additional vulnerabilities in online contexts. It further calls for specialised research and tailored interventions within educational settings to ensure risks related to gambling are not overlooked.

## Summary

- Secondary school teachers perceive gambling to be a risk for both typically developing and SEN students but attribute greater risks from gambling to SEN students than typically developing students.
- Teachers view gambling‐related risks as secondary to other risky behaviours such as alcohol and drug use, for both typically developing and SEN students, which participants associated with limited knowledge of gambling‐related harms.
- Training programmes may help enhance secondary school teachers' understanding of gambling‐related risks to aid early intervention and prevention efforts.

## 1 Introduction

Adolescent gambling has emerged as a significant public health concern (Armitage 2021). In the United Kingdom, 27% of 11‐ to 17‐year‐olds engaged in gambling activities in the past year (Gambling Commission 2024). Although recognised as a high‐risk behaviour, gambling is often perceived by parents and teachers as less problematic compared with other risky behaviours such as smoking and alcohol use (Roberts et al. 2022). Adolescents, prone to risk‐taking, impulsivity and thrill‐seeking (Wilber and Potenza 2006), are particularly susceptible to gambling due to cognitive immaturity (Emond and Griffiths 2020). They may misunderstand probabilities or have illusions of control over outcomes (Emond and Griffiths 2020). Additionally, their executive functioning is not fully developed, further increasing risk‐taking and impulsivity (Blakemore and Choudhury 2006).

Furthermore, the ubiquitous use of smartphones exposes adolescents to gambling advertisements on social media, making online gambling appear attractive (Emond and Griffiths 2020). These advertisements reinforce the perception that gambling is socially acceptable among peers (Parrado‐Gonzalez and Leon‐Jariego 2020) and portray it as an opportunity to socialise (Wilber and Potenza 2006). Additionally, entities not legally classified as gambling but with gambling‐like mechanisms, such as loot boxes, are highly accessible to adolescents (Meduna et al. 2020). Contemporary games that integrate randomness through the purchase of loot boxes introduce adolescents to less regulated gambling (Roberts et al. 2022). The ease of access to gambling, especially online in‐game gambling, has led to a notable increase in adolescent gambling (Armitage 2021), significantly raising the likelihood of their engagement in these activities (Emond and Griffiths 2020).

Recently, gambling awareness charities identified various indicators of gambling‐related harms among students, such as school absenteeism, withdrawal, mood and behaviour changes, debt, unexplained funds or possessions, preoccupation with gambling and anxiety (Ygam 2022). Research indicates that adolescents who gamble experience emotional distress, low mood, guilt and disruption of daily life. They may also face social and financial consequences, including social isolation, relationship challenges and debt (Livazović and Bojčić 2019; Raisamo et al. 2012). Interestingly, Raisamo et al. (2012) found that adolescents are less likely to report financial consequences of gambling, possibly due to having limited access to independent funds. However, financial consequences may manifest later in life, as financial harms are more commonly observed in adults (Browne 2018). Despite the evident signs and severe consequences, gambling often remains a ‘hidden addiction’ as it lacks overt physical signs that are typically seen in risk‐taking behaviours (Singh and Malik 2023). This invisibility reduces teachers' and parents' ability to detect gambling risks among adolescents, contributing to a low level of awareness (Splevins et al. 2010).

Adolescents with special educational needs (SEN), defined as having a learning difficulty or disability that requires special educational provision beyond that available to their peers due to significantly greater difficulty in learning than most others of a similar age or barriers to utilising facilities in mainstream education (Department for Education & Department for Health 2015), may be more susceptible to problematic gambling behaviours than their typically developing (TD) peers (Taylor et al. 2014). For instance, previous research suggests that some adolescents with learning difficulties may experience a greater risk of gambling problems compared to their TD peers (Parker et al. 2012) due to a higher prevalence of erroneous gambling‐related cognitions (GRCs) (Taylor et al. 2014). These GRCs include misunderstandings about odds, probability and control over outcomes (Hardoon and Derevensky 2002). Literacy and numeracy difficulties associated with SEN may contribute to cognitive biases related to probability and mathematical odds, thereby increasing the risk of gambling‐related harms among SEN students (Parker et al. 2012). For example, some research indicates that adolescents diagnosed with attention deficit hyperactivity disorder (ADHD) may experience a high propensity for problematic gambling behaviours, potentially linked to differences in executive functioning and behavioural inhibition (Faregh and Derevensky 2011).

Concerns regarding vulnerabilities related to risky behaviours among SEN students have prompted research into teachers' influence on student behaviour (Roberts et al. 2022). Teachers, through their daily interactions with students and observations, are well‐positioned to notice signs of risky behaviours, including gambling (Ruzek et al. 2016). In fact, evidence suggests teachers play a more pivotal role than parents in identifying gambling behaviours (Sansanwal et al. 2014). However, this depends partially on awareness and confidence (Sansanwal et al. 2014), and teachers often struggle to accurately assess the prevalence of gambling among students (Castrén et al. 2016). A UK study (Roberts et al. 2022) found that only a minority of secondary mainstream teachers accurately knew current gambling prevalence rates, indicating a general lack of awareness. Nonetheless, over 50% of teachers identified common characteristics associated with gambling‐related harms, a finding that contrasts with previous research and may reflect recent increased media coverage of gambling (Roberts et al. 2022). Further, studies in both Canada (Derevensky et al. 2013) and the United Kingdom (Roberts et al. 2022) showed that secondary school teachers ranked gambling as less serious than other adolescent risky behaviours such as drug use, alcohol abuse and violence.

Notably, teacher training typically does not include coverage of gambling behaviours, and teachers have expressed limited interest in this topic, perceiving other high‐risk behaviours as more pressing (Castrén et al. 2016). Despite 38% of teachers witnessing students actively gambling, implementation of school prevention programmes remains a low priority (Derevensky et al. 2013). The UK curriculum's insufficient coverage of gambling awareness may lead to infrequent discussions and lack of teacher confidence in addressing it (Roberts et al. 2022). However, enhancing teachers' knowledge of gambling‐related risks can reduce adolescent gambling, emphasising the need for more comprehensive teacher training and resources (Tani et al. 2021).

In January 2025, over 1.7 million pupils (19.5%) in England were identified as having SEN. The majority were educated in mainstream schools, including almost all pupils receiving SEN support and 56.2% of those with an Education, Health and Care Plan (EHCP) (Department for Education 2025). As a result, mainstream teachers play an increasingly central role in ensuring the support and inclusion of SEN students (Humphrey and Lewis 2008). Research has identified higher rates of some risky behaviours, including gambling, among some students with SEN compared with TD students (Taylor et al. 2014). This underscores the need for teachers to better comprehend gambling behaviours among SEN students. However, existing research primarily quantifies teachers' awareness and understanding of gambling behaviours among TD students, whereas the experiences of SEN students remain largely unexplored (Parker et al. 2012). To date, no studies have specifically investigated teachers' perceptions of gambling behaviours in SEN populations.

Given the limited research in this area, alongside evidence indicating elevated rates of gambling‐related risks among SEN students, it is imperative to examine teachers' awareness of these risks within this population. As schools play a critical role in safeguarding, prevention and early intervention, teachers' awareness of gambling‐related risks may have significant implications for students' well‐being, health and social development. Research in this area could inform teacher training and support the development of targeted school‐based interventions aimed at mitigating gambling‐related harms and promoting the well‐being of SEN students. Therefore, this study aims to investigate mainstream secondary school teachers' awareness of gambling‐related risks in SEN students, including whether they perceive these risks differently for SEN students relative to their TD peers. The primary research question is as follows: What is mainstream secondary school teachers' awareness of gambling‐related risks among SEN students? A secondary research question is as follows: How do teachers perceive the severity of gambling‐related risks relative to other risky behaviours?

## 2 Methods

### 2.1 Participants

Participants were recruited through existing professional networks, employing purposive sampling (Cresswell and Plano Clark 2011). Eligible participants were current teachers in mainstream secondary schools in the United Kingdom. Exclusion criteria included being retired, on a career break, not being a teacher, having personal experiences with gambling or being negatively affected by family, friends or partners' gambling behaviour. Inclusion and exclusion criteria were outlined in the Participant Information Sheet, which was provided to all teachers who expressed interest in the study. In addition, researchers confirmed participants' eligibility against these criteria immediately prior to commencing the interviews.

The study sample comprised 15 teachers (11 females and 4 males), aged 23–57 years (*M* = 40.33, SD = 11.86), from mainstream secondary schools across the United Kingdom. Research suggests saturation typically occurs within 12 interviews, making 15 interviews sufficient to obtain rich data (Guest et al. 2006). Participants' teaching experience ranged from 1 to 27 years (*M* = 12.07, SD = 8.74). They taught a variety of subjects including Geography, English, Science, Mathematics, Physical Education, Spanish, Music and Religious Education. Nine participants completed the Postgraduate Certificate in Education (PGCE) training, with others trained directly via school or Qualified Teacher Status (QTS) routes.

### 2.2 Design and Procedure

An exploratory approach with an interpretive perspective was adopted for its flexibility and capacity to produce rich data (Braun and Clarke 2021). This methodology enables a nuanced understanding of participants' experiences by connecting their meanings to the wider context of the data (Braun and Clarke 2021).

This study was part of a bigger project looking at teachers' perceptions of gambling‐related harms among secondary school students. The study adhered to the BPS Code of Human Research Ethics (Oates et al. 2021) and complied with the General Data Protection Regulations (GDPR 2018). Ethical approval was granted by the Faculty of Science and Technology Ethics Committee at Bournemouth University (Ethics ID: 52583), and written informed consent was obtained from all participants. Semi‐structured interviews were conducted by three researchers (M.D., J.H., and B.V.) to capture participants' perspectives (McGrath et al. 2019) and provided both structure and flexibility to explore emerging topics (Adeoye‐Olatunde and Olenik 2021). The interview schedule (Supporting Information: Section 1) explored teachers' views and experiences of gambling behaviours in TD and SEN students, including how they felt gambling compared with other risky behaviours and any training they had received in relation to gambling. All interviews were conducted online, audio‐recorded and transcribed verbatim between 8 January and 10 March 2024 and lasted 45–60 min. Afterward, participants received a debrief form and an Amazon voucher for £20.

### 2.3 Data Analysis

The data were analysed using Braun and Clarke's (2021) inductive six‐phase reflexive thematic approach, chosen for its suitability in deeply exploring participants' individual experiences and the researcher's active role in knowledge creation. This method values the researcher's subjective interpretation and allows for continuous revisiting and refinement of the analysis (Braun and Clarke 2021).

Initially, the lead author (M.D.) familiarised herself with the data by transcribing the interviews manually, which facilitated deep immersion within the dataset (Byrne 2021). To protect participant confidentiality, pseudonyms were assigned in accordance with the BPS Code of Human Research Ethics guidance on confidentiality (Oates et al. 2021). Then, initial brief but precise codes were generated by working systematically through the data. These codes were then used to generate themes, where different codes with shared meanings were combined. Potential themes were reviewed for relevance to the research question. Themes and sub‐themes were then defined, named and organised into the theme table (Supporting Information: Section 2). A proportion of the interviews were second coded by another researcher. A reflexive diary was maintained throughout the analysis (Supporting Information: Section 3).

### 2.4 Reflexivity

Among the three researchers who conducted the interviews, one had worked in a gambling clinic and supported SEN college students, and the other two had experience in SEN primary schools. This background provided insights into the complexity of gambling addiction, the potential vulnerabilities and risk factors that SEN students may face and school dynamics and teacher interactions with SEN students. However, the researchers' prior experiences working with SEN students might have meant they were more likely to pay attention to teachers' experiences that linked with their own experiences. To maintain objectivity, the analysis was checked by two independent researchers: one with expertise in gambling‐related harms and the other with expertise in resilience research in children and young people.

## 3 Results

**FIGURE 1:**
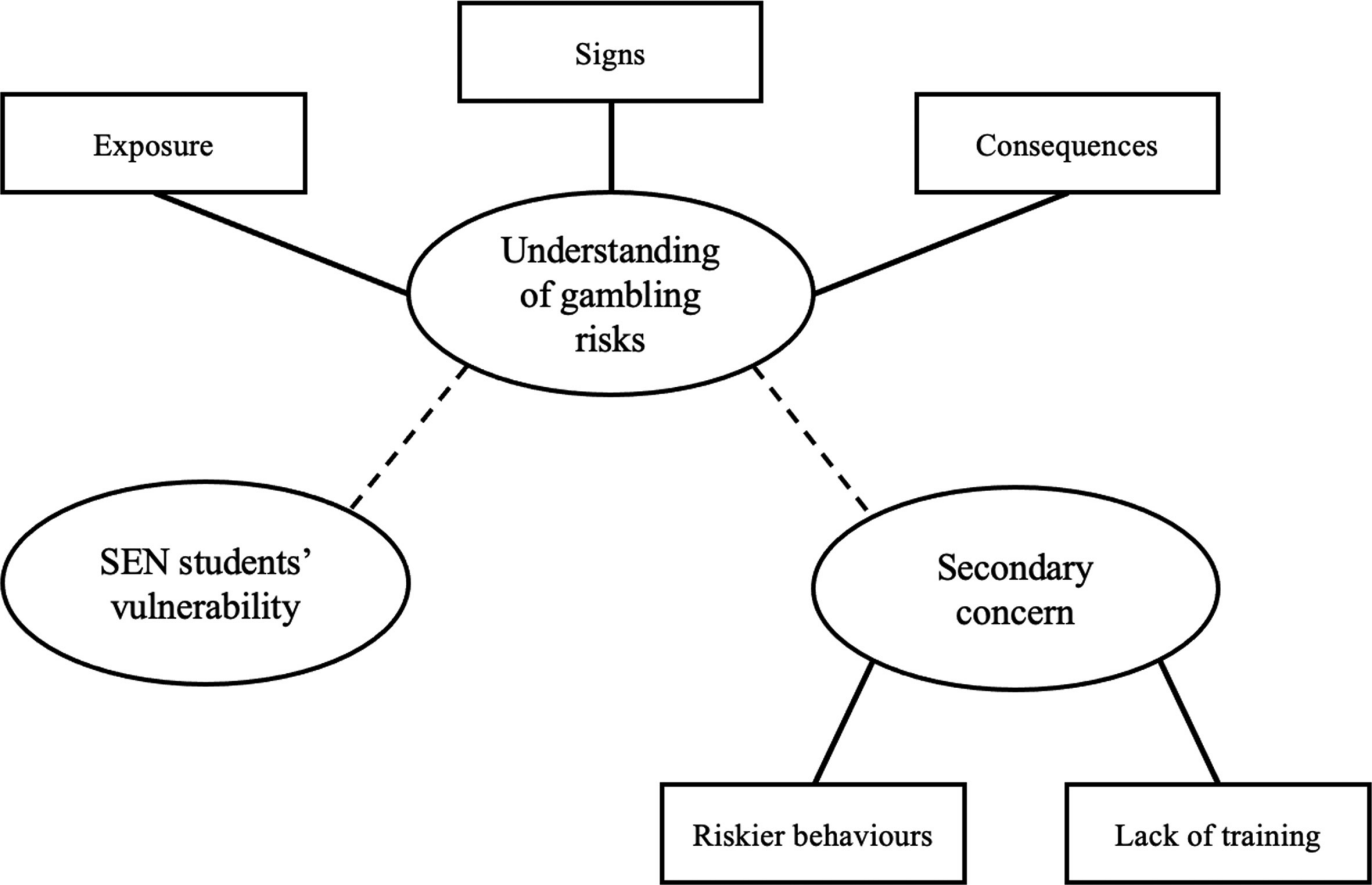
Themes and subthemes identified.

Three main themes were identified (Figure 1). The first theme, ‘Understanding of gambling‐related risks’, captures the extent of teachers' knowledge about gambling‐related risks and included three sub‐themes: ‘Exposure’, ‘Signs’ and ‘Consequences’. The second theme, ‘SEN students' vulnerability’, explores teachers' opinions regarding the vulnerability of SEN students compared with their TD peers. The third theme, ‘Secondary concern’, emphasises that gambling is not the primary focus for teachers when it comes to addressing risky behaviours and included two sub‐themes, ‘Riskier behaviours’ and ‘Lack of training’.

### 3.1 Understanding of Gambling‐Related Risks

This theme highlights teachers' awareness of students experiencing gambling‐related harms. Teachers expressed concerns about students' level of exposure to gambling and could identify its signs and consequences. Many participants felt their knowledge was similar for both TD and SEN students. Three sub‐themes were identified within this theme: ‘Exposure’, ‘Signs’ and ‘Consequences’.

#### 3.1.1 Exposure

Participants noted high levels of exposure to gambling materials, with technology increasing this risk. All thought students would ‘probably see gambling related stuff daily on their phones’ (P11). Teachers thought phones were the main medium via which students would have access to gambling materials, particularly through social media: ‘I think that social media would probably be the main way that I would think they were exposed to gambling’ (P3).

Participants expressed how accessible gambling adverts are on social media due to the sheer number available: ‘The amount that students can constantly see, like gambling advertisement on TV, on their phones’ (P6). Gambling advertisements were also mentioned by participants in relation to sports: ‘a lot of people are football fans, for example, so when they are playing football, they have advertising about gambling’ (P12).

#### 3.1.2 Signs

Participants identified various signs of gambling‐related risks in students, with several highlighting behavioural changes. They saw behaviours including ‘arrogant’, ‘cocky’, ‘secretive’, ‘aggression’, ‘quiet’, ‘withdrawn’, ‘anxious’ and ‘obsessive’ as worrying if they were not the students' ‘normal behaviours’ (P8). Participants also cited increased gambling knowledge and discussion as suspected signs of gambling:

Usually, it's more about what kids say, so if they said something that was a bit strange and indicated that they had more knowledge than they should of gambling. (P4)

Several participants also felt an increase in expensive items would be a sign of gambling: ‘They might start spending things, or, you know, on more flashy coats or equipment, watches that kind of thing. They do like a good Apple Watch’ (P2). Finally, participants believed that ‘being obsessive about their mobile phone’ (P13) might be a good indicator of student gambling, linking to beliefs that students were exposed to gambling through social media. However, alongside recognising several signs of gambling‐related risks, participants also recognised that gambling may be easily hidden: ‘it'd be quite hard to spot to be honest unless they talked about it openly’ (P2).

#### 3.1.3 Consequences

Consequences refer to how gambling would affect students in the future. The main consequence mentioned was students facing addiction in later life: ‘They'll probably be more likely to have an addiction or have issues controlling the gambling that they do’ (P7). In addition, many participants highlighted students' lack of understanding about money and future debt issues: ‘They will actually get into debt and have money issues later on in life and may be less able to manage some of their money’ (P5).

Some participants focused on the immediate future consequences of gambling, such as poor school performance:

Feeling too tired to be in school, so kind of missing school will probably be a factor or unable to concentrate or loss of performance or homework going down because of the time and tiredness involved. (P9)

### 3.2 SEN Students' Vulnerability

Participants believed SEN students were more susceptible to gambling‐related risks than their TD peers. They cited issues related to their awareness of students' needs and the difficulties they perceive students encounter, such as perception and executive function limitations in SEN students, suggesting that these students may struggle to access their previous knowledge of risks:

So, there is a vulnerability there in their perception, forget the impulsivity side of ADHD, for example. But, if they have an executive function limitation, I can see that … even if they were asked to pause and think about it, they might still not be able to access their knowledge and memories about the previous risks and consequences and outcomes that they've had. (P2)

Participants also focused on SEN students with specific diagnoses, including Autism and ADHD, whom they saw as becoming more obsessive and fixated with gambling:

Special educational needs they're more likely to become so obsessive over something so you know where an addict might be obsessed perhaps that obsession would be far more pronounced in the student with special educational needs. (P1)

I feel like they could be particularly vulnerable to this concept of, especially the Casino stuff, like the poker and that sort of thing, like thinking that they can like master a skill and getting very fixated on that. (P11)

Participants also suggested that SEN students may be more vulnerable to gambling‐related risks due to processing difficulties that could affect their understanding of consequences:

SEND is to do with their processing and understanding and stuff, they won't always necessarily understand the consequences or be able to process the consequences. (P4)

Additionally, teachers suggested that SEN students might lack understanding of socially acceptable behaviour compared to TD students:

They are a little bit more naive, especially when you've got children who haven't learned societal norms and values the way that you know neuro typical people are socialised. (P7)

### 3.3 Secondary Concern

Teachers acknowledged gambling‐related risks but did not prioritise them compared with other behaviours they deemed riskier for both TD and SEN students. Because of insufficient training on gambling‐related risks, teachers struggled to grasp the full extent of these risks.

#### 3.3.1 Riskier Behaviours

Teachers believed a range of other behaviours were riskier than gambling, due to the physical consequences. Some identified drugs as the riskiest behaviour, due to the risk of death:

Whereas gambling is not likely to straight up kill you, the consequences might be dangerous, and you might get yourself in a really bad situation with people who owe your money but just like one instance of gambling is not likely to harm you as much as one bad batch of drugs, for example. (P7)

Other behaviours that participants viewed as being riskier included alcohol use, online behaviours, vaping and being part of a gang:

I think drug use probably alcohol is probably the next one outside of school that are probably the biggest risks, that I think students are susceptible. (P6)

Online. Definitely. It's quite a regular occurrence really. In various forms of who they interact with, who they send images and videos, who they meet up with based on online interactions, whether it puts them in dangerous positions. (P9)

‘I always think if you are in a gang for me if you are in a gang, I think that is one of the top ones’ (P12). Some participants touched upon gambling being ‘one of the lowest risky behaviours’ (P13), commenting that they were not currently overly concerned about it. Although participants were aware of the risks of gambling, it was not their primary concern compared to other risky behaviours.

#### 3.3.2 Lack of Training

Participants' limited concerns around gambling‐related risks can be at least partly attributed to their lack of training. None had received any gambling‐specific training during their teacher training or teaching positions: ‘There's no topic that we don't cover really, apart from actually gambling’ (P2). This contrasted with the provision of training for other risky behaviours, resulting in teachers feeling more confident in discussing these compared to gambling: ‘As it currently stands with the level of training, I would feel fairly unconfident in terms of dealing with gambling as opposed to other risky behaviours’ (P13). The lack of training also meant that teachers felt they lacked sufficient knowledge of the extent of gambling‐related risks for students: ‘I don't know enough about it to be absolutely certain there's no risk to students in my school’ (P9). Teachers felt they would benefit from training to enable them to support and guide students: ‘I want more training to feel like I'm giving them like the most relevant information’ (P11).

This contrasts with training teachers had received on other risky behaviours, which incorporated a focus on how signs of these behaviours may present differently in SEN students:

It's never been something that we are trained on [how gambling impacts SEN students], and we are trained on that with other risky behaviours, so we're taught about how signs for an autistic student who's using drugs might differ from other kids or how SEN kids might display signs of abuse in a different way. (P11)

## 4 Discussion

This study aimed to explore mainstream secondary school teachers' awareness of gambling‐related risks among students with SEN. It investigated teachers' perceptions of gambling behaviours among SEN students compared with their TD peers. Analysis revealed three themes, ‘Understanding of gambling‐related risks’, ‘SEN students' vulnerability’ and ‘Secondary concern’. Teachers exhibited awareness of gambling‐related risks for both TD and SEN students, with some participants expressing concerns that certain SEN students may experience higher risk in relation to learning, communication or support needs. However, teachers viewed gambling‐related risks as secondary compared to other risky behaviours, for both TD and SEN students, likely due to their lack of knowledge of gambling.

Our study highlighted teachers' concerns about adolescents' exposure to gambling and demonstrated that they were aware of gambling‐related risks in both TD and SEN students. This finding contrasts with previous research suggesting teachers have low awareness regarding gambling‐related risks among TD students (Roberts et al. 2022). However, previous studies assessed awareness based on identification of prevalence rates and DSM‐IV criteria for pathological gambling (Derevensky et al. 2013; Roberts et al. 2022). In contrast, this study explored a broader spectrum of understanding, including exposure, signs and consequences of gambling, which may possibly help explain the greater levels of awareness reported by participants. Additionally, the use of qualitative methods may have yielded more nuanced and comprehensive insights compared with surveys (Mwita 2022).

Our findings echo common knowledge of gambling‐related risks among students. In line with recent research, participants noted frequent exposure to gambling content via advertisements on social media (Emond and Griffiths 2020). Moreover, our participants reported recognising several signs of gambling harm in students, such as behavioural and mood changes, increased material possessions and fixation with gambling, in line with advice from gambling support charities (Ygam 2022). Additionally, research indicates that financial harm from gambling is more prevalent in adults than children (Browne 2018). Similarly, our participants associated adolescent gambling with potential financial difficulties in adulthood, emphasising their perceptions of the need for early intervention (DiClemente et al. 2000). Teachers' reported awareness of gambling‐related risks appears heightened compared with previous research, possibly influenced by increased media attention to gambling issues (Roberts et al. 2022) and public and political discussions on gambling‐related harm in the United Kingdom, including the publication of the gambling White Paper in April 2023.

In this first study investigating teachers' awareness of gambling‐related risks among SEN students, participants frequently viewed SEN students as potentially more susceptible to gambling‐related risks compared with their TD peers, in line with evidence from Canada that rates of gambling are higher among male SEN students than those without learning disorders (Parker et al. 2012; Taylor et al. 2014). Further research is needed to explore how gambling‐related risks are experienced differently across diverse SEN populations, including potential gender differences and contextual factors that may shape these experiences. This may help inform targeted and more inclusive school‐based gambling interventions. Teachers in our study attributed SEN students' perceived vulnerability to gambling‐related harms to executive function limitations and processing difficulties commonly observed in inclusive classrooms (Humphrey and Lewis 2008). These insights advocate for additional research into inclusive practices used in UK schools and whether these practices can be adapted to prevent engagement in gambling among SEN populations.

However, although our participants perceived SEN students as being at heightened risk of gambling‐related harms, our findings should be interpreted with caution. Although teachers' perceptions are essential for understanding how risk, professional challenges and support needs are conceptualised within school settings, they should not be interpreted as suggesting that SEN students are inherently vulnerable to gambling‐related harms. Rather, participants' accounts reflect perceptions formed by personal and professional experiences, as well as the broader social context surrounding SEN. It is therefore important to acknowledge the potential for a deficit‐based model to unconsciously shape interpretations about SEN students' strengths, weaknesses and diverse online experiences.

We found that teachers prioritise addressing other risky behaviours, particularly drug and alcohol, while reporting insufficient training on gambling risks, in line with previous research (Roberts et al. 2022). Risky behaviours are likely a particularly significant issue in relation to SEN students, given the volume of training required to work effectively with such students (Kang and Martin 2018), and the SEN‐specific training provided in relation to other risky behaviours. Previous studies suggest that teachers' lack of concern stems from inadequate coverage of gambling relative to other risky behaviours in teacher training (Castrén et al. 2016) and the absence of educational resources on gambling (Wybron 2018). Participants reported limited coverage of gambling‐related risks during teacher training or within teaching positions, which they perceived as contributing to their lack of confidence in responding to gambling‐related risks compared with other risky behaviours. Research shows that training teachers about gambling‐related risks can reduce students' participation in gambling activities (Tani et al. 2021), underscoring the necessity for targeted training to effectively mitigate gambling‐related harm among both TD and SEN students.

### 4.1 Strengths, Limitations and Future Research

Qualitative interviews facilitated in‐depth insight into teachers' awareness of gambling‐related risks among SEN students. This approach captures detailed data, enhancing understanding of the research issue (Mwita 2022). Furthermore, this study's unique focus on teachers' perceptions of SEN students' potential vulnerability to gambling‐related risks sets an important foundation for future research. Although these findings reflect participants' observations and experiences rather than objective characteristics of SEN students, they highlight areas that warrant further investigation. For instance, future studies could involve SEN students to explore how societal or educational context may affect their experiences related to gambling; this would lay the groundwork for developing non‐stigmatising support strategies to reduce gambling‐related harm among SEN students.

Although this study enhances our understanding of teachers' awareness of gambling‐related risks among SEN students, it has some limitations. First, data were collected from only 15 participants, which limits transferability to the wider population. In particular, we recruited only four male teachers—important as perceptions of gambling may differ by gender (Wong et al. 2013). Further research with more male teachers is needed. Second, only qualitative data were collected. Although this was justified by the aims of the study, it did not account for other variables that could further explain how teachers perceive gambling, such as self‐efficacy. Future research could extend these findings by incorporating standardised measures to assess additional variables. Second, purposive sampling may have led to over‐recruitment of teachers with a particular interest in gambling, potentially excluding those with lower awareness and therefore limiting diversity and representativeness in the sample. As a result, the findings of this study may reflect the views of a more accessible group of teachers rather than the perspectives of the broader target population of teachers. Additionally, providing a recruitment incentive might have shaped participation patterns. Incentives may attract participation from participants who are more motivated by compensation. This can introduce self‐selection bias, elicit socially desirable responses or lead participants to provide false characteristics to meet the eligibility criteria of the study (Head 2009) and in turn affect data quality. However, even teachers who were interested in gambling expressed a lack of confidence in addressing gambling‐related risks among students, underscoring the need for training across teachers.

Furthermore, because of its exploratory nature, this study considered SEN as a broad category, encompassing a wide range of learning and support needs (Krischler et al. 2018). Future research on specific SEN, such as Autism Spectrum Conditions, may help to develop greater insight into individual and contextual factors associated with gambling‐related vulnerability.

### 4.2 Practical Implications

Although exploratory in nature, this study offers important insights into teachers' awareness of gambling‐related risks among SEN students. The widespread accessibility of gambling underlines the need to address its impact on both TD and SEN students. Teachers' accounts also suggest that there may be value in developing and implementing training programmes to enhance teachers' understanding of gambling‐related risks, particularly among SEN students, which may aid early intervention and prevention of gambling‐related harm across secondary schools. These programmes could also correct misconceptions about the severity of gambling as a risky behaviour, allowing teachers to address gambling‐related concerns as confidently as they address other risky behaviours.

Moreover, this study contributes to the evidence base regarding perceptions of vulnerability to gambling‐related harms among SEN students (Parker et al. 2012; Taylor et al. 2014), as reflected in teachers' concerns regarding potential elevated risk. Although these findings derive from a small qualitative sample, they underscore the potential value of tailored and flexible interventions around gambling education, as SEN students may benefit from alternative delivery and support modes. Implementing such interventions may allow teachers to provide responsive support that reflects the diverse needs and experiences of SEN students to proactively reduce the risk of gambling‐related harm.

## 5 Conclusion

To the best of our knowledge, the present study was the first study to explore mainstream teachers' awareness of gambling‐related risks among SEN students. Teachers reported increased awareness of gambling‐related risks in both TD and SEN students but perceived this issue as less serious due to inadequate training compared to other risky behaviours. These findings corroborate previous research on teachers' misconceptions of the seriousness of gambling and warrant a need for the implementation of teacher training programmes on gambling‐related risks. Concerns about SEN students' potential vulnerability to gambling‐related risks were noted, linked to perceived characteristics commonly associated with SEN. Importantly, these findings are derived from a small exploratory study and depict teachers' perceptions rather than inherited attributes of SEN students. They should therefore be interpreted with caution to avoid endorsing deficit‐oriented framing of SEN students. Further research is needed on the development and evaluation of teacher training to inform school‐based interventions that are accessible, non‐stigmatising and tailored to the heterogenous needs and experiences of SEN students aimed at mitigating gambling‐related harm.

## Author Contributions

**Megan Dobson:** formal analysis, writing – original draft, writing – review and editing, investigation, validation. **Emily Arden‐Close:** supervision, formal analysis, writing – review and editing, methodology, validation, conceptualization. **Constantina Panourgia:** conceptualization, funding acquisition, writing – review and editing, methodology, supervision, project administration, resources.

## Funding

This work was part‐funded by GambleAware. GambleAware is a grant‐making charity using best practice in commissioning, including needs assessment, service planning, evaluation and outcome reporting to support effective, evidence‐informed, quality‐assured prevention of gambling harms. Guided by a public health model, GambleAware commissions integrated prevention services on a national scale and in partnership with expert organisations and agencies, including the UK National Health Service, across three areas of activity: universal promotion of a safer environment (primary); selective intervention for those who may be ‘at risk’ (secondary); and direct support for those directly affected by gambling disorder (tertiary). The authors alone are responsible for the views expressed in this article, which do not necessarily represent the views, decisions or policies of the institutions with which they are affiliated (www.about.gambleaware.org).

## Ethics Statement

All procedures followed were in accordance with the ethical standards of the Faculty of Science and Technology Ethics Committee, Bournemouth University, the British Psychological Society Code of Conduct for Ethics Oates et al. 2021) and the Declaration of Helsinki 1975, as revised in 2000. Ethical approval was granted by the Faculty of Science and Technology Ethics Committee at Bournemouth University (Ethics ID: 52583).

## Consent

All participants provided written informed consent to take part in the study, which included agreement to be interviewed, to have the interviews recorded, and for their anonymised data to be used in future research projects, including publications, reports and presentations.

## Conflicts of Interest

The authors declare no conflicts of interest.

## Supporting information




**Data S1:** Supporting information.

## Data Availability

The data that support the findings of this study are available from the corresponding author upon reasonable request.
