# Supplementary material for: Teachers' Perceptions of Gambling‐Related Risks Among Students With Special Educational Needs: An Exploratory Study
Source: Child Care Health Dev. 2026 Jul 14;52(4):e70320. doi: 10.1111/cch.70320 (PMC13367255; doi:10.1111/cch.70320)
Supplement: Supplementary file 1 — Data S1: Supporting information. [file CCH-52-e70320-s001.docx]

**SUPPLEMENTARY MATERIALS**

**SECTION 1: Interview Schedule**

Before starting the interview, it will be important to check that participants are currently working in a mainstream school in the UK and they have not had experiences gabling harms or/and have not negatively been affected by their family’s/partner’s/ friends’ gambling behaviours

Demographic information:

- Age
- Gender
- Subject taught (and whether SEND/mainstream if applicable)
- Ages taught
- Date of qualifying as a teacher
- Number of years teaching experience
- Type of teacher training received: 4-year degree; PCGE; school-based training
- 1. What do you think gambling is?
- 2. In what ways do you believe your students are exposed to gambling?
  - How often do you believe your students are confronted with gambling materials?
- 3. What would you perceive to be an early sign/indicator that a student is gambling?
  - What about if your student had Special Educational Needs/Disabilities (SEND)? Would the signs be any different?
- 4. What do you believe are the consequence of engaging in gambling behaviours as an adolescent?
  - How might this change for a student with SEND?
  - Do you think students with SEND experience gambling harms differently to those without SEND?
  - If so, how?
  - Why?
- 5. How would you define risky behaviour in adolescent years?
  - Can you list any?
  - What are the most risky behaviours in which adolescents engage?
- 6. How would you compare gambling to the other risky behaviours provided?
- Do the signs of gambling look different compared to the signs of other risk behaviours?
  - Is gambling more or less severe than other risky behaviours?
- Why so? Is this the case for students with SEND too?
- 7. If you were concerned that a student is at risk of gambling, what would you do?
- 8. If you were concerned about a student with SEND, would you take the same actions?
- 9. Are you currently aware of any gambling prevention strategies for your students? If so, what are they?
- 10. What would you consider appropriate intervention/prevention measures?
- Do you think there are any barriers to student engagement in current prevention programmes or strategies?
- If so, what are they?
- 11. Do you feel that students with SEND can benefit from current prevention programmes or strategies the same way that non-SEND students can?
  - What barriers do you perceive that students with SEND face when learning about youth problem prevention strategies?
  - How might intervention/prevention measures require alterations for students with SEND?
  - Are there any modes of delivery for prevention programmes or strategies that you feel work better for students with SEND? If so, what are they?
  - Why do you feel these modes benefit SEN students?
- 12. How comfortable would you be in engaging in preventative measures against gambling harms regarding your students?
  - How would that differ if intervening with a student with SEND?
- 13. Was gambling awareness covered within your teacher training?
- Has gambling awareness been covered within your teaching post, for example through INSET days?
- If yes, what was your experience of this training?
  - How does this compare to other risky behaviours such as drugs/alcohol or violence?
  - Would you be open to further training?
    - What would incentivise you?
- 14. How would you currently feel about talking to your pupils around gambling risks?
  - How might your approach alter if discussing with a student with SEND?
  - If teacher hasn’t received any training: What about if you had received training and it was introduced as a part of the curriculum? Would you feel more comfortable to open discussions with your students?
  - If not, why?

**SECTION 2: Themes and Sub-themes Table**

| Theme | Sub-theme | Description | Supporting Quotations |
| --- | --- | --- | --- |
| Understanding of gambling risks | Exposure | The exposure to gambling material was understood to be high. Participants spoke about students being exposed to gambling risks daily/weekly, particularly through adverts on social media or TV. | “There's some cultures within the school as well as my demographic of kids generally, their parents tend to be more into gambling and that kind of thing” (P2)  “So actually, if they're watching football every week on the telly, they'll get it weekly” (P2)  “I think that social media would probably be the main way that I would think they were exposed to gambling” (P3)  “They’re exposed to gambling, from quite an early age, is through online apps” (P5)  “I think it's online gambling. I think there's so much there's so much that they can get hold of on their phones. There's so much in especially on sports betting in terms of like football” (P6)  “The amount that students can constantly see, like gambling advertisement on TV, on their phones” (P6)  “Gambling is so mainstream, there’s ads on regular TV. There's, you know, there's billboards. So, a lot I would imagine” (P7)  “So I would guess, for some students really, really regularly, and for other students, much less so based on how much they've engaged with that sort of material before” (P8)  “I think definitely could be daily or weekly, I think quite easily” (P9)  “I think they probably see gambling related stuff daily on their phones” (P11)  “I think nowadays is quite easy to be exposed to gambling, for example, especially lot of people are very football fans, for example. So when they are playing football, they have advertising about gambling” (P12)  “You will have adverts about that. So, I think nowadays is daily” (P12)  “The main one will be social media. So, through the adverts that they watch online” (P13)  “I imagine probably weekly at least and I guess it depends what kind of media they're accessing” (P13)  “Using social media every day I would assume that they would be exposed to it” (P14)  “I feel that exposure is massive now compared to what anything, you know, what I've experienced, and I think they're exposed through the television. They're exposed through social media” (P15)  “So I absolutely think daily because actually, on the television now, we're seeing it daily” (P15) |
|  | Signs | Participants noted that an increase in expensive items, talking about gambling, a change in behaviour, and being obsessed with their phones were signs of gambling. | “Children would display secretive and obsessive kind of behaviours around something that they’re gambling on” (P1)  “They might start spending things, or, you know, on more flashy coats or equipment watches that kind of thing. They do like a good apple watch. But it'd be quite hard to spot to be honest unless they talked about it openly” (P2)  “Talking about kind of how much money they've won, or how much money they've lost and talking about kind of staying up late, maybe” (P3)  “Usually, it’s more about like what kids say, so like if they said something that was a bit strange and umm like indicated that they had more knowledge than they should of gambling” (P4)  “so you can usually tell because there’s like a shift in behaviour” (P4)  “I think you know, if you're trying to, if you're trying to hide this, then I think aggression and anger at first” (P5)  “Sort of the way they act around school, sort of being quite arrogant or cocky depending on what they're bringing into school. So, things like what they're wearing, if they got a new phone, new watch, all that sort of things” (P6)  “All those signs that we would worry about for any students where things just aren't their normal behaviours, places where they are, not their normal selves - very up, very down, very quiet, very absent” (P8)  “Signs of being withdrawn or up late at night, or obsessing over their phone” (P9)  “That they've suddenly they've got some, more money, um expensive things that they can afford for themselves” (P10)  “Maybe even a change in personality, you know. Little bit bit worried bit anxious. Maybe those kind of emotional or behavioural changes” (P10)  “Change of behaviour, I think that will be a very good sign” (P12)  “You can see the lack of sleep that and all of that is going to affect negatively to their to their school work” (P12)  “Asking their mates for money, or borrowing money all the time, and being obsessive about their mobile device” (P13)  “Probably that they have suddenly got um nice clothes or new trainers” (P14)  “Maybe just there'd be a bit of a personality change” (P15) |
|  | Consequences | Participants expressed a range of consequences of gambling. The main consequences mentioned were being less financially responsible and experiencing addiction in the future. | “I think if you're successful, I think there's a huge risk to carrying it on later in life” (P2)  “I suppose again it becomes it really winds up with addiction doesn't it” (P3)  “I think it harms their understanding of the value of money in the future” (P4)  “They will actually get into debt and have money issues later on in life and maybe less able to manage some of their money” (P5)  “They don't see the importance of money, or they don't, they don’t see it as a necessity, and rather than something that's actually really important everyday life” (P6)  “They'll probably be more likely to have an addiction or have issues controlling the gambling that they do” (P7)  “They'd be obviously less financially responsible” (P7)  “Feeling too tired to be in school, so kind of missing school will probably be a factor or unable to concentrate or loss of performance or homework going down because of the time and tiredness involved” (P9)  “I think that would be long time sort of issues with maybe budgeting. Um, trusting of money and people, er could even be yeah, sort of high spend, so high spending rate” (P10)  “Once you get to gamble, you get addicted” (P12)  “Another thing is mental health, I think, is a big issue of that, self like low self-esteem is going to be, anxiety, it can make you change the personality of how you normally are” (P12)  “Obviously the obvious one is debt and then that leading into issues with getting into borrowing money” (P13)  “You can get yourself into debt and potentially in trouble with the law” (P14)  “I suppose it starts an addiction, addictive type of behaviour” (P14) |
| SEN students’ vulnerability |  | Participants suggested that SEN students were more vulnerable to gambling risks compared to TD students. This was due to deficits associated with SEN such as processing difficulties and hyper-fixation. | “Special educational needs they're more likely to become so obsessive over something so you know where an addict might be obsessed perhaps that obsession would be far more pronounced in the student with special educational needs” (P1)  “So actually, there is a vulnerability there in their perception, forget the impulsivity side of ADHD, for example. But actually, if they have an executive function limitation, I can see that the perception, even if they were asked to pause and think about it, they might still not be able to access their knowledge and memories about the previous risks and consequences and outcomes that they've had, so that might be a factor as well” (P2)  “SEND is to do with their processing and understanding and stuff, they won’t always necessarily understand the consequences, or be able to process the consequences” (P4)  “Students with autism might be more vulnerable to this, and not understanding the implications of what they're viewing, and how potentially, how harmful is so they might be affected by it” (P6)  “I think also vulnerable children, um they're more likely to be swayed by the adults in their lives doing it, or the adverts promising certain things because they are a little bit more naive, especially when you've got children who haven't learned societal norms and values the way that you know neuro typical people are socialised” (P7)  “Terms of some of our autistic students. Kind of obsessive stuff with our autistic students, and perhaps some perception issues with some of our autistic students would be areas that would concern me” (P8)  “Our SEND students would be in many ways at greater risk. So yes, I guess if everything else remained the same, then probably their way of understanding what was happening and putting actions into place to change if they wish to would be less successful independently” (P8)  “They haven't got the numeracy skills, maybe not realise how much money is, is going out” (P10)  “Some of them are like they get very hyper fixated on things, especially often quite niche computer-based hobbies, that's quite common” (P11)  “I feel like they could be particularly vulnerable to this concept of, especially the Casino stuff, like the poker and that sort of thing, like thinking that they can like master a skill and getting very fixated on that” (P11)  “I think some of the students who have SEND and lack certain social skills and stuff like that might be more likely to be peer pressured into gambling and getting involved in things that they don't fully understand” (P11)  “They could be more obsessive of something and more fixated” (P12)  “I think they're going to do it because they haven't got a sensibility of what is right and what is wrong” (P12)  “They often don't appreciate the kind of the social norms and a lot of non-SEND students will know that gambling isn't a socially acceptable kind of thing for someone to do” (P13)  “Possibly a lack of awareness, possibly a lack of understanding” (P14)  “SEND students depending on their need, that could be a wider issue or a bigger problem because they don't have that understanding” (P14)  “I think it that it's vulnerability. It's their ability to discern and part of that is linked with intellect for sure” (P15) |
| Secondary concern | Riskier behaviours | Participants expressed that gambling was not their primary concern due to other behaviours being riskier. Other behaviours that were deemed to be riskier included drugs, vapes, alcohol, and gangs. | “I suppose with something like county lines, I suppose that it would be that these children are targeted, and they have been taken advantage of, and they are getting in with all sorts of people that might do them harm, and I'd be worried that they wouldn't, you know, they don't understand things enough to be able to manage all of that” (P3)  “I think with drugs, obviously you might think about that was a risk to death, the risk of death, kind of risks of extreme changes in behaviour again, with risks of addiction. So, I would say that the risks are higher” (P3)  “I would say, like I don’t think it’s really something that we’re overly concerned about at the moment, I don’t know whether that’s maybe because we wouldn’t be like necessarily aware if they’re gambling because that’s usually something that you’d do on your phone not, you’d have to go somewhere and they’re not 18 yet, whereas like, they’re very much capable of getting like vapes from their mum’s bag or something or very much capable of fighting with their peers” (P4)  “I think drug use probably alcohol is probably the next one outside of school that are probably the biggest, the biggest risks, that I think students are susceptible” (P6)  “Substance abuse would be the riskiest” (P7)  “Whereas gambling is not likely to straight up kill you, the consequences might be dangerous, and you might get yourself in a really bad situation with people who owe your money. But just like one instance of gambling is not likely to harm you as much as one bad batch of drugs, for example” (P7)  “Online. Definitely. It's quite a regular occurrence really. In various forms of who they interact with, who they send images and videos, who they meet up with based on online interactions, whether it puts them in dangerous positions” (P9)  “I would probably still say it's less than other forms of um risky behaviours” (P10)  “I would say, the most widespread risk that we currently see those vaping” (P11)  “Drugs, I would say we have had a lot of issues with drugs, knives as well. Yeah. yeah, gambling isn't. It's not seen as such a big problem” (P11)  “I always think if you are in a gang for me if you are in a gang. I think that is one of the top one” (P12)  “Most prevalent is vaping and most significant is drugs” (P13)  “Whereas something like vaping and drugs, I think the significance of it is much higher, because you've only got to do it once, and it can have a massive impact on you” (P13)  “Gambling is one of the lowest risky behaviours” (P13) |
|  | Lack of training | The reason for participants lack of concern towards gambling risks was related to their lack of training. Participants highlighted that they have never received any training in relation to gambling, and this has had an effect on their knowledge and confidence with addressing gambling risks. | “I must say I’m not aware of gambling being a subject for safeguarding at all in my recent memory at all, no” (P1)  “There's no topic that we don't cover really, apart from actually gambling” (P2)  “I've just done my advanced safeguarding and my designated safeguarding lead training, and it was interesting that actually, gambling didn't come up in there, either” (P2)  “I think you know it's nothing, I've had experience with” (P3)  “I don't remember any kind of anything really coming up when in teacher training about gambling” (P3)  “Not much at the moment, but that’s because I haven’t had any training” (P4)  “I looked through all the personal development lessons that we have to give, and there isn’t one on gambling” (P5)  “I would well at the moment without any information I would just follow the PowerPoint and it would probably be lacking” (P5)  “Not within sort of the safeguarding training, sort of looking for maybe potential signs that students are like I said a lot previously in terms of if students are at risk” (P6)  “I think because it is so accessible and because it's so socially acceptable. Um, alcohol is very similarly, socially acceptable, but we've got all these safeguards. Why is gambling different?” (P7)  “Not as a main topic, it possibly is half mentioned but not whereas ones like County Lines” (P8)  ‘I don't know enough about it to be absolutely certain there's no risk to students in my school” (P9)  “With having had no experience of it, or what looks like, what kind of slot machines or roulette type kind of games. I wouldn't know very much at all” (P9)  “I do think that the gambling online it's probably rising, but it is not seen as a problem. So like in all of our safeguarding training, our CPD that we get, not once as gambling ever been mentioned to staff” (P11)  “I want more training to feel like I'm giving them like the most relevant information” (P11)  “I’m in a gang or haven’t got much money, poverty in the poverty line, I will be more comfortable to try to talk to them and if they tell me I've got a gambling addiction, actually it will set me aside” (P12)  “I will need training for both for special needs and no special needs because we haven't got nothing, so yeah” (P12)  “As it currently stands with the level of training, I would feel fairly unconfident in terms of dealing with gambling as opposed to other risky behaviours” (P13) |

**SECTION 3: Reflexive Diary**

11/03/24

Following transcription, I coded the data which has helped me to gain a deeper understanding of participants experiences. I have started to group initial codes from the data that related to each other. Potential concepts focus on SEN students being more at risk of gambling harms. Teachers are aware that exposure to gambling material is high due to increase in advertisement and phone use. Participants were able to list what they think is a sign or consequence of gambling for students with little difference between SEN and TD students. Another concept that is clear is the fact that participants view other risky behaviours as being more serious compared to gambling risks. The range of risky behaviours listed differs from each participant.

13/03/24

Reading over initial codes highlights participants understanding of why SEN students may be more at risk to gambling harms. Particular deficits in processing, hyper-fixation and socially acceptable behaviour come to mind. Over-arching this is the fact that teachers believe that SEN students are unable to differentiate right from wrong, therefore would be unable to interpret gambling as a socially unacceptable behaviour. An initial thought that comes to mind when reading codes is that teachers are aware of gambling, aware of the risks associated with it however they are not concerned given that they view other risky behaviours to be more serious. These thoughts have led to many different possibilities of sub-themes, although I am unsure how to group them currently.

20/03/24

Through focusing on the grouping of initial codes, preliminary themes began to stand out. Most of the participants focused on SEN students being more vulnerable to gambling risks with a sub-theme of this being that the vulnerability is due to deficits associated with SEN. Another aspect that is clear is teachers’ knowledge of gambling risks. This can be shown through their understanding of the level of exposure, signs, and consequences of gambling. The final theme that comes to mind is teachers’ concerns about gambling. There are ideas of gambling not being their primary concern and other behaviours being more concerning.

23/03/24

It is proving difficult to refine themes and understand the relationship between them. Through going back through initial codes and noting down key ideas that stand out, it is helping me to understand the data beneath the surface. Some initial themes and sub-themes were noted but still need to be refined so that they are clear. Another idea that is frequently mentioned is the lack of training in relation to gambling risks. All participants have never had any experience of training around gambling risks which is clearly linked to their lack of concern. It appears that their current understanding of gambling risks is through their own research or general understanding of risky behaviours.

26/03/24

When going through key ideas again, I have picked out three potential themes. One relating to teachers’ knowledge of gambling risks, another relating to SEN students’ vulnerability and finally one relating to teachers lack of concern. I started to group some codes under these themes with exposure, signs and consequences coming under teachers’ knowledge. The main reason for teachers perceiving SEN students to be more vulnerable related to deficits associated with SEN, which I can see being used as a sub-theme. The lack of concern theme relates to teachers’ opinions on other risky behaviours. I also understand this lack of concern to be related to the fact that teachers have not received training about gambling risks.

02/04/24

I have started to refine themes and sub-themes even further by pulling out quotes from the data to start compiling a theme table. I decided to name the first theme as ‘Understanding of gambling risks’ with the sub-theme’s ‘Exposure’, ‘Signs’, and ‘Consequences’. The second theme I named ‘Vulnerability to gambling risks’ with the sub-theme ‘Deficits associated with SEN’. The final theme I named ‘Secondary concern’; this was because it was clear from the data that gambling risks are not teachers’ primary concern. The sub-themes for this theme were ‘Other risky behaviours’ and ‘Lack of training’. This refinement of themes and sub-themes made it easier for me to start going through the data and choose the key quotes related to each one.

05/04/24

I have been continuing to take quotes from the data and put them in the theme table however I am not feeling fully satisfied with some of the names for the themes and sub-themes. I have decided I am satisfied with the first theme and its sub-themes ‘Understanding of gambling risks’. I was unsure on the name of the second theme and decided to change it to ‘SEN students’ vulnerability’, I feel this will be clearer for the reader. The final theme ‘secondary concern’, I decided to change the name of one of the sub-themes from ‘other risky behaviours’ to ‘riskier behaviours’, as again I feel like this is clearer. These changes have left me satisfied with my themes and sub-themes as I feel that it is representative of the participants experiences with gambling risks. I will now move on to producing a thematic map and the report.

12/04/23

I decided to return to the theme ‘SEN students’ vulnerability’ and remove its one sub-theme. After consideration and some advice, it made more sense to have ‘SEN students’ vulnerability’ as a stand-alone theme given that most main themes generally have two sub-themes. Following this change, I altered the thematic map and part of my results section.
